# The development of a brief screener for autism using item response theory

**DOI:** 10.1186/s12888-019-2333-y

**Published:** 2019-11-04

**Authors:** Caroline Mårland, Gitta Lubke, Alessio Degl’Innocenti, Maria Råstam, Christopher Gillberg, Thomas Nilsson, Sebastian Lundström

**Affiliations:** 1 Centre for Ethics Law and Mental Health (CELAM), Institute of Neuroscience and Physiology, Sahlgrenska Academy, University of Gothenburg, Gothenburg, Sweden; 2 Department of Forensic Psychiatry, Sahlgrenska University Hospital, Gothenburg, Sweden; 3 Department of Psychology, University of Notre Dame, Notre Dame, USA; 4 Department of Clinical Sciences Lund, Lund University, Lund, Sweden; 5 Gillberg Neuropsychiatry Centre, Institute of Neuroscience and Physiology, Sahlgrenska Academy, University of Gothenburg, Kungsgatan 12, SE-411 19 Göteborg, Sweden

**Keywords:** Autism, Screening, A-TAC

## Abstract

**Background:**

Brief screening instruments focusing on autism spectrum disorder (ASD) that can be administered in primary care are scarce; there is a need for shorter and more precise instruments. The Autism–Tics, AD/HD and other Comorbidities inventory (A-TAC) has previously been validated for ASD reporting excellent validity. This study aims to determine the psychometric properties of each item in the ASD domain (17 items) in the A–TAC using item response theory (IRT), and thereby construct and validate a short form that could be used as a screening instrument in the general population.

**Methods:**

Since 2004, parents of all 9-year-old Swedish twins have been invited to participate in a telephone interview in the Child and Adolescent Twin Study in Sweden (CATSS). The CATSS is linked to the National Patient Register (NPR), which includes data from in- and outpatient care. Data on ASD (A-TAC) collected in CATSS were compared with diagnoses from the NPR. Diagnoses that had been made both before (previous validity) and after (predictive validity) the interviews were included. The sample was divided into a developmental sample and a validation sample. An IRT model was fitted to the developmental sample and item parameters were used to select a subset of items for the short form. The performance of the proposed short form was examined in the validation sample by the use of receiver operation characteristic curves.

**Results:**

Four items which were able to discriminate among individuals with more autism traits were deemed sufficient for use in the short form. The values of the area under the receiver operating characteristic curve for a clinical diagnosis of ASD was .95 (previous validity) and .72 (predictive validity).

**Conclusions:**

The proposed short form with 4 out of the original 17 items from A-TAC, showed excellent previous validity while the predictive validity was fair. The validity of the short form was in agreement with previous validations of the full ASD domain. The short form can be a valuable screening instrument in primary care settings in order to identify individuals in need for further assessment and for use in epidemiological studies.

## Background

Autism Spectrum Disorder (ASD) is characterized by onset during the early developmental period and manifested by deficits in social communication, social interaction and restricted, repetitive behavior [1]. Emerging evidence suggests that ASD traits are continuously distributed in the general population, where individuals with an ASD diagnosis represent the extreme end [2, 3]. A diagnostic assessment of ASD, consistent with the prevailing diagnostic systems DSM-5 and ICD-10, includes developmental history as well as assessment of social and communication skills and repetitive and stereotyped behaviors [1, 4]. It may also include medical history, physical examination, systematic considerations about coexisting conditions to establish differential diagnoses, observations in different environments and taking the child’s needs, strengths, skills, and impairments into account [5]. In addition, assessment in multidisciplinary teams (e.g. child and adolescent psychiatrists, psychologists and speech and language therapists) is generally recommended [6].

The absolute majority of children referred for diagnostic assessments have been identified via less specialized instances such as mandatory developmental check-ups, or through parental concerns leading to primary care visits. In addition to observation, the initial visit could encompass broadband and/or narrowband screening instruments. For ASD, a number of instruments are available such as the Modified Checklist for Autism in Toddlers (which includes 23 items, reported sensitivity/specificity: .87/.99) [7]), the Autism Spectrum Screening Questionnaire (27 items [8], .62/.90 [9]), the Social Communication Questionnaire (40 items [10], .85/.75 [11]), the Social Responsiveness Scale (65 items, .85/.75 [12]), and the Autism–Tics, ADHD and other Comorbidities inventory (A–TAC, 17 items, .96/.88 [13]). These instruments range between 17 and 65 questions and even though they might be considered to be brief, there is a need for shorter and reliable instruments. An initial screening should be broad and tap into the whole child and adolescent psychiatric field in order to allocate the child to the most suitable assessment service where a full clinical investigation is conducted.

Classical test theory has widely been used to determine scale reliability by analyzing the scale as a whole with little regard to the underlying distribution or item specific characteristics. In classical test theory, the reliability of a scale is connected to the correlations between all items that constitute the scale. Cronbach’s coefficient alpha has widely been used as a measure of reliability and the alpha value can be improved by adding items or by enhancing the average inter-item correlation [14]. Item response theory (IRT), however, offers an opportunity to examine each individual item in a scale and enhance reliability by identifying items with maximum precision. In IRT, an individual’s response to an item is assumed to be explained by an underlying (latent) trait. An IRT analysis offers an assessment of an item’s psychometric quality in relation to its position on the latent trait continuum, and its discrimination abilities on the same continuum. Thus IRT has an advantage in the ability to guide scale reduction with retained reliability and measurement precision [15, 16].

To the best of the author’s knowledge only one article has employed IRT on a screening instrument for ASD. In this article, the Social Responsiveness Scale was reduced from 65 to 16 items while maintaining high reliability [17]. However, the sample consisted of 21,426 individuals where >50% had a registered ASD diagnosis and the ‘controls’ were mainly siblings without a diagnosis of ASD but, most likely, with an elevated ASD symptomatology. Taken together this primarily generates information about the sensitivity but very little about the specificity, rendering the usage of the instrument limited as a screener in primary care where the entire continuum of autistic traits is represented.

The A–TAC has been part of the nationwide Child and Adolescent Twin study in Sweden (CATSS) for over a decade, and has been validated using classical test theory [13, 18–22]. The purpose of this paper is to advance our understanding of the autism domain in the A–TAC, by taking the growing needs for a short and time-effective ASD screening instrument with high precision and minimal response burden into consideration. More specifically the aims of this study were to: (a) determine the psychometric properties of each item in the ASD domain in A-TAC using IRT, (b) construct a short form that could be used as a screening instrument in the general population, and (c) validate the proposed short form and determine cut-off values.

## Methods

### Sample

The sample was retrieved from CATSS, which is an ongoing longitudinal study with the aim of assessing somatic and mental health problems during childhood. A detailed description of the CATSS can be found elsewhere [23]. Beginning in 2004, parents of all Swedish 9-year-old twins (born 1st July 1995 onwards) are invited to participate in a telephone interview in connection with their twins 9th birthday (during the first 3 years of the study 12-year-olds, born 1st of July 1992–30st of June 1995, were also included). The CATSS has a response rate of (> 70%) and small differences between responders and non-responders concerning the prevalence and correlates of neurodevelopmental disorders when compared to the National Patient Register (NPR). For instance the prevalence of ASD-diagnoses in responders has been reported to be 0.84 and 0.95% in non-responders. Furthermore, among non-responders, 1.8% had been prescribed psychopharmacological treatment for ADHD as compared to 1.4% of the responders [23].

Each individual born, or receiving a citizenship, in Sweden is given a personal identification number which renders linkage across registers possible. The CATSS sample was merged with the NPR, which contains best estimate specialist diagnoses assigned according to the International Classification of Diseases ninth (ICD-9) and 10th (ICD-10) revisions [4, 24]. Since 1987 the NPR includes information about all assigned diagnoses in the psychiatric inpatient care and information from the outpatient care has been included since 2001. In Sweden, regional guidelines for diagnosing ASD suggest a five step process: 1) a comprehensive interview with the patient, incorporating patient history; 2) scrutiny of medical journals; 3) an examination of the patient’s function in day-to-day life; 4) a psychological assessment, and 5) a medical evaluation [25]. Furthermore, the ASD diagnoses in NPR have been subjected to validation. Idring et al. (2012) [26] reported an agreement of 96% between medical records and registered diagnoses when comparing several registers. Diagnostic data were obtained from the NPR by searching for ICD-9 and ICD-10 codes that correspond to an ASD diagnosis. The retrieved codes were 299.0, 299.8, 299.9 (ICD-9) and F84.0, F84.1, F84.5, F84.9 (ICD-10).

The sample was retrieved in May 2018 and included a total of 30,898 subjects (12,315 boys and 12,065 girls aged 9 years and 3349 boys and 3169 girls aged twelve years) out of which 93 individuals were excluded due to missing data. Furthermore, the sample included 427 individuals (M:F ratio 2.5:1) that had been assigned an ASD diagnosis in the NPR.

### Measure

A–TAC [18] is a fully structured broad-band screening instrument originally designed for large-scale epidemiological research. The instrument is a comprehensive and easy-to-administer parental interview that has been used in the CATSS and is administrated by laymen over the phone. It consists of 96 items out of which 17 constitute the ASD domain. The items are based on the diagnostic criteria of pervasive developmental disorder (autistic disorder, 299.00) phenotype in DSM-IV [27], clinical experience and clinical features that have been captured by other available screening instruments, such as Asperger Syndrome Screening Questionnaire [8, 9], the Asperger syndrome Diagnostic Interview [28] and the instrument ‘5–15’ that has been validated for those age ranges [29]. The ASD domain (α = .86) contains three theoretically defined modules; Language (6 items, α = .66), Social interaction (6 items, α = .77) and Flexibility (5 items α = .70) [23]. Each module begins with the statement “The essential aspect of each question is whether the problem/peculiarity has been pronounced compared to peers during any period of life”. Each item from the ASD domain and their corresponding item number and module are reported in Table 1. All items are coded:” No″ scored as 0,” Yes, to some extent” scored as .5, and” Yes” scored as 1. The ASD domain has been validated both cross-sectionally and longitudinally. Cross-sectional validations report Areas Under the Receiver Operating Characteristics Curve (AUC) for autism ranging between .88–.96 [13, 18], the interviews were conducted at ages 6–19. Longitudinal validations report AUCs ranging from .81–.91 [20, 22], all interviews were conducted at age 9 or 12 and then followed up at ages 10–20. In addition, a Spanish version of the ASD domain has been independently validated and the result indicated excellent psychometric properties [19]. Finally, the test-retest intraclass correlation was reported to be .84 and the κ-value was .59 for a screening cut-off and 1.0 for a cut-off corresponding to a clinical proxy of ASD [21].

**Table 1:** The ASD domain in A–TAC

Module	Item	Item content
Language	H34	Was his/her language development delayed or doesn’t he/she speak at all?
	H35	Does he/she have difficulties sustaining a conversation?
	H36	Does he/she like to repeat words and expressions or does he/she use words in a way that other people find strange?
	H37	Does he/she have difficulties with games of make-believe or does he/she imitate others considerably less than other children?
	H38	Does he/she talk in too high a pitch or too quietly?
	H39	Does he/she have difficulties keeping “on track” when telling other people something?
Social interaction	I40	Does he/she have difficulties expressing emotions and reactions with facial gestures, prosody, or body language?
	I41	Does he/she exhibit considerable difficulties interacting with peers?
	I42	Is he/she uninterested in sharing joy, interests, and activities with others?
	I43	Can he/she only be with other people on his/her terms?
	I44	Does he/she have difficulties behaving as expected by peers?
	I45	Do other people easily influence him/her?
Flexibility	J46	Does he/she get absorbed by his/her interests in such a way as being repetitive or too intense?
	J47	Does he/she get absorbed by routines in such a way as to produce problems for him/herself or others?
	J48	Has he/she ever engaged in strange hand movements or toe-walking when he/she was happy or upset?
	J49	Does he/she get obsessed with details?
	J50	Does he/she dislike changes in daily routines?

### Statistical analyses

#### Item response theory

In the first step we tested the assumption of unidimensionality which implies that a single underlying trait or factor accounts for a substantial majority of the covariance among the items of a scale. This was examined by using an exploratory factor analysis (EFA) with principal factor analysis and a promax rotation to account for the correlation between items. In order to avoid capitalization on chance the analysis was conducted in a randomly selected sample of approximately 1% (*N* = 295). We used a scree plot to determine the point of inflexion (where the slope of the line changes dramatically) and concluded that the demand of unidimensionality was met. The factor loadings and the scree plot are available as supplementary material (Additional file 1). Taken together the results from the scree plot and the EFA were considered to represent a sufficiently unidimensional scale.

For this study, the sample was randomly divided into a developmental and a validation sample. The developmental sample was used to fit an IRT model and select a subset of items based on the estimated IRT item parameters. The validation sample was used to confirm the performance of the selected scale with receiver operating characteristics (ROC) curves.

The developmental sample included 15,408 subjects out of which 210 had a registered autism diagnosis in the NPR. Given the large dataset, the response frequencies in each response category seemed sufficient in order to utilize all three response categories in the IRT analysis of the ASD domain (please see Additional file 2: Table S2). A graded response model was used to analyze the difficulty and discrimination parameters of the ASD domain. For each item, this model features a difficulty parameter, usually denoted as *b,* which identifies the location on the latent trait continuum (here: severity of autism) where the probability of endorsing a response category is 0.5. The second item parameter is the discrimination parameter, usually denoted as *a*, which indicates how well the items distinguish between individuals at different levels of the latent trait (e.g., a low estimated value indicates lower discrimination along the autism severity scale).

The item information functions from the IRT model in the developmental sample were used to select five items (at least one item from each module was included to maintain content coverage) with high discrimination in the extreme end of the autism continuum. Next, local dependence (usually a subset of items that have very similar content) was examined as these may cause inflated slope estimates. All item parameters from the IRT-model were therefore manually examined for content-similarity [15].

In the final part of our study we used the validation sample, including 15,490 subjects out of which 217 had a registered diagnosis in the NPR, to calculate ROC curves to determine the AUC. The AUC indicates how well an instrument can discriminate between a true positive and a false positive disorder for all possible values on a parameter and it also yields information about the sensitivity and specificity values for each scale step. The AUC can be used as a measure of validity, an AUC of .5 indicates random prediction, .60–.70 poor validity, .70–.80 fair, .80–.90 good and > 0.90 indicates excellent validity [30]. The possible short form of the ASD domain was used as an independent predictor and the clinical diagnoses from the NPR was used as a dependent variable. All analyses on the validation sample were stratified by when the first diagnosis of autism was listed in the NPR, before (previous) or after (predictive) the A–TAC interview. A registered diagnosis the same year as the A–TAC interview was considered as a listed diagnosis before the A–TAC interview since the clinical assessment could have begun before the age of 9 or 12. The analyses were also conducted in a total group which included the whole sample.

The IRT analyses were conducted in the STATA 15 software and all other analysis were performed in the SPSS software package, version 22.0.

#### Ethical considerations

The CATSS and the linkage to the NPR have received ethical approval from the Karolinska Institute ethical review board (Dnr 02–289 and 2010/507–31/1).

## Results

### Item response theory

The item parameter estimates and the corresponding standard error from the graded response model are reported in Table 2. The slope estimates ranged from 1.15 to 3.4, while the difficulty parameter estimates at the first threshold (b_1_, yes to some extent) ranged from 1.11 to 2.81 and at the second threshold (b_2_ = yes) the range was 2.32 to 3.57. The result indicates that the ASD domain can discriminate among subjects in the far end of the autism trait continuum and that the higher response categories are only endorsed for subjects who have a higher than average level of ASD (i.e. theta is greater than 0).

**Table 2:** Item parameter estimates and standard errors from the graded response model in the developmental sample

Module	Item	*a*	SE	*b*_1_	SE	*b*_2_	SE
Language	H34	1.15	.04	2.45	.07	2.97	.09
	H35	2.54	.1	2.28	.04	2.98	.06
	H36	2.02	.07	2.16	.04	2.78	.06
	H37	1.96	.07	2.38	.05	3.0	.07
	H38	1.2	.04	2.22	.06	3.57	.10
	H39	1.80	.05	1.74	.03	2.69	.06
Social Interaction	I40	2.73	.12	2.44	.05	3.01	.07
	I41	3.29	.11	1.75	.02	2.39	.04
	I42	2.36	.1	2.52	.05	3.27	.08
	I43	2.41	.07	1.83	.03	2.62	.05
	I44	3.4	.11	1.64	.02	2.42	.04
	I45	1.28	.03	1.11	.03	2.61	.06
Flexibility	J46	1.98	.06	1.62	.03	2.48	.05
	J47	2.64	.09	2.11	.03	2.81	.06
	J48	1.46	.06	2.81	.08	3.31	.10
	J49	2.32	.06	1.52	.02	2.41	.04
	J50	2.13	.06	1.42	.02	2.32	.04

*N*= 15,408

*a*= item discrimination, SE = standard error, *b*_*1*_ & *b*_*2*_ = item difficulty

### Short form item selection

Both items I41 (Does he/she exhibit considerable difficulties interacting with peers?) and I44 (Does he/she have difficulties behaving as expected by peers?) displayed high discrimination values as well as similar location of the difficulty parameter. Given the statistical and wording similarities, item I41 was removed from the final analysis. Four items, H35, I40, I44 and J47 were selected as candidates for the short form due to high discrimination values.

### Validation of the short form

The AUC and corresponding sensitivity and specificity for each scale step are reported in Table 3. The AUC ranged between .72–.95 depending on age at diagnosis. Two cut-offs, (a) yielding a high sensitivity but lower specificity (> = 0,5) and (b) yielding a lower sensitivity but higher specificity (> = 1.5) are suggested to be used to identify ASD in children.

**Table 3:** Previous and predictive validity of the ASD domain short form

	Previous	Predictive	Total
AUC	.95	.72	.81
Scale step	sens/spec	sens/spec	sens/spec
0.5	.949/.899	.519/.898	.681/.902
1	.782/.964	.349/.963	.512/.967
1.5	.654/.984	.248/.983	.401/.986
2	.50/.99	.155/.989	.285/.992
2.5	.372/.995	.07/.994	.184/.995
3	.295/.997	.039/.996	.135/.997
3.5	.128/.999	.016/.998	.058/.999
4	.09/.999	.008/.999	.039/1.0

*N* = 15,490

Previous and predictive validity: Area under the Curve (AUC) and sensitivity/specificity

Please see the supplementary material for the number of true positive, false positive, true negative and false negative, as well as positive predictive value, negative predictive value and diagnostic odds ratio for each proposed cut-off value for the ASD domain short form (Additional file 3: Table S3)

## Discussion

The primary aim of this study was to conduct an IRT analysis in order to construct a valid and reliable short form of the ASD domain in A–TAC. Our main finding is that four questions can satisfactorily discriminate among subjects in the far end of the autism trait continuum, especially in children below the age of nine. The short form reported excellent previous validity and the results were in agreement with a previous validation of the full ASD domain (17 items) [22].

The applications of the short form can be multi-faceted, even though a short form cannot be used as a diagnostic instrument. Its strengths lie in quickly identifying those individuals that would benefit from a further assessment of ASD in order to facilitate early identification of symptoms, and by extension, early diagnosis and intervention. ASD affects approximately 1% of the population [31] and early interventions can give developmental gains, such as enhanced cognitive function and adaptive behavior [32]. Today, it is still unclear if general screening of ASD should be incorporated in primary care [33], however, some organizations recommended general screening for toddlers [34, 35]. In this instance a check-up will most likely cover several areas, such as food intake, communication, language development, sleeping habits, motor skills and somatic examination. Therefore, it is important that relevant general screening instruments for ASD are time-effective and brief as well as validated in general population settings. Sweden, as well as several other countries, provides regular check-ups for children in primary care settings. Robins [36] reported that out of 21 children diagnosed with ASD only four were identified by health-care providers as suitable for further psychiatric assessment. Taken together this suggests that screening instruments may indeed aid the identification of ASD in health care settings even in the presence of trained professionals conducting developmental surveillance. The ASD domain short form reported excellent validity before the age of 9 or 12 and can therefore be used when concerns are raised during a regular check-up, primary care visits or in elementary school settings.

The predictive validity was fair while the sensitivity values were rather low (.519 and .248) for the proposed cut-off scores. This indicates that the short form may not be optimal as a screening instrument for older age-groups in clinical settings. However, Arvidsson et al. [37] reported a substantial decrease in autism symptom score in A-TAC for individuals who were diagnosed with ASD at ages 7–12, but with a less explicit decrease for the language module. Future research could include an examination of age-specific cut-off values and consider if language deficits should be further highlighted during assessment of ASD in older children.

In research settings brief and time-effective instrument with minimal response burden can be a valuable resource in large-scale epidemiological studies. Primarily, where the goal is to determine prevalence figures and when there is no need for an assessment that encompasses the broader phenotypic variance. Furthermore, in low-income countries where societal resources may be limited, brief and easy to access instruments that are free of charge are needed. The A–TAC is an open access instrument that can be downloaded in Swedish or English from the Gillberg Neuropsychiatry Centre website, http://gnc.gu.se [38], and it is also included as an appendix in Larson et al. [13].

The primary strength in the present study is that it consists of a large population-based sample, and its linkage to the NPR, which contains best-estimate clinical diagnoses. However, the result in this study should be considered in light of some limitations. First, the scores from A-TAC were retrieved in connection with the 9th or 12th birthday and the short form should be used with awareness of possible differences between age-groups. However, the questions in A-TAC are asked in a “whole-life” frame and the respondents are asked to consider if a specific problem has been pronounced compared to peers and the questions are modelled around the DSM-IV definition of Autistic Disorder (299.00) [27], which are general descriptions that vary greatly depending on the developmental level and chronological age. Secondly, the validation of the short form was conducted in a sample of respondents who had completed all 96 A-TAC items, thus item order bias cannot be ruled out. However, the Cubo et al. [19] validation only consisted of the ASD-domain and came to very similar conclusions as previous articles as to why the effect of order is most likely not a major problem. A future validation of the short form should nevertheless be completed in an independent sample. Thirdly, differences in response pattern in possible sub-groups, such as boys and girls, were not examined, on the other hand the reported male-female sex ratio were at par with previous publications [39]. Finally, the sample was based on twins and it has been argued that twins may have an increased risk for ASD [40, 41]. However, this assumption has not been confirmed in large-scale epidemiological studies [42–44] or within the CATSS [31].

## Conclusions

The ASD domain in A–TAC has the ability to discriminate among subjects in the far end of the autism trait continuum. The proposed short form, with 4 out of the original 17 items, showed excellent previous validity while the predictive validity was fair. The ASD domain short form can be a valuable instrument as a screener in primary care settings in order to identify individuals in need of further assessment and in epidemiological studies.

## Supplementary information

**Additional file 1: **Exploratory factor analysis. Result from the EFA: **Figure S1.** shows the scree plot, **Table S1.** includes the un-rotated factor patterns for a single factor solution and **Table S2.** includes the rotated factor patterns for five factors.

**Additional file 2: Table S2.** Response frequencies. The table presents the response frequencies for each item in the developmental sample.

**Additional file 3: Table S3.** Cross tables, positive predictive value, negative predictive value and diagnostic odds ratio. Includes cross tables that present the numbers of true positive, false positive, true negative and false negative in the previous, predictive and total group for the ASD domain short form. The positive predictive value, negative predictive value and diagnostic odds ratio for the proposed cut-off values in the ASD domain short-form are also presented.

## Data Availability

The datasets are available on reasonable request from the corresponding author at sebastian-lundstrom@neuro.gu.se

## Abbreviations

ASD: Autism spectrum disorders
A-TAC: the Autism–Tics, ADHD and other Comorbidities inventory
AUC: Area under the receiver operating characteristics curve
CATSS: the Child and Adolescent Twin Study in Sweden
EFA: Exploratory Factor Analysis
ICD: International Classification of Diseases
IRT: Item response theory
NPR: National Patient Register
ROC: Receiver operating characteristics
