# Supplementary material for: The development of a brief screener for autism using item response theory
Source: BMC Psychiatry. 2019 Nov 4;19:337. doi: 10.1186/s12888-019-2333-y (PMC6829932; doi:10.1186/s12888-019-2333-y)
Supplement: Supplementary file 1 — Additional file 1: Exploratory factor analysis. Result from the EFA: Figure S1. shows the scree plot, Table S1. includes the un-rotated factor patterns for a single factor solution and Table S2. includes the rotated factor patterns for five factors. [file 12888_2019_2333_MOESM1_ESM.docx]

**Exploratory factor analysis (EFA)**

The following figure and tables includes the result from the EFA in approximately 1 % of the total sample. Figure 1 shows the scree plot and table 1 includes the un-rotated factor patterns for a single factor solution. Table 2 includes the rotated factor patterns for the maximum amount of five factors, following the Keiser-Guttman criterion.

**Figure 1** Scree plot


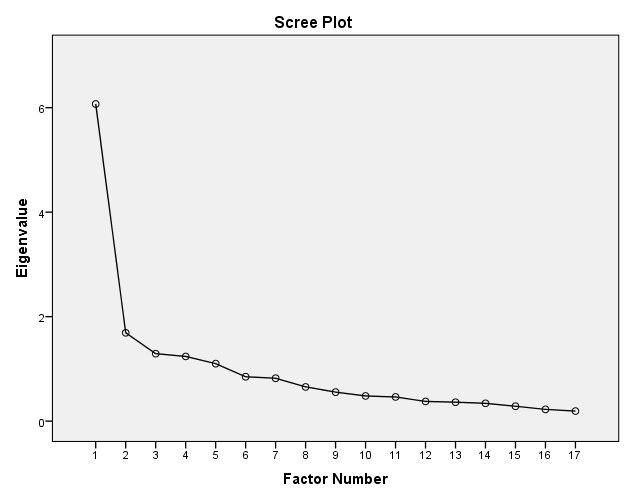


**Table 1** Single factor solution for the ASD domain by using principal axis factoring.

| Module | Item |  | Factor 1* |
| --- | --- | --- | --- |
| Language | H34 |  | .203 |
|  | H35 |  | .637 |
|  | H36 |  | .556 |
|  | H37 |  | .472 |
|  | H38 |  | .305 |
|  | H39 |  | .397 |
| Social Interaction | I40 |  | .615 |
|  | I41 |  | .684 |
|  | I42 |  | .575 |
|  | I43 |  | .596 |
|  | I44 |  | .666 |
|  | I45 |  | .226 |
| Flexibility | J46 |  | .685 |
|  | J47 |  | .780 |
|  | J48 |  | .377 |
|  | J49 |  | .730 |
|  | J50 |  | .685 |

* Un-rotated factor pattern.

N = 295.

**Table 2** Factor loadings for the ASD domain by using the principal axis factoring with promax rotation.

|  |  | | Two factors | |  | | Three factors | | | |  | | Four factors | | | | |  | | Five factors | | | | | |  |
| --- | --- | --- | --- | --- | --- | --- | --- | --- | --- | --- | --- | --- | --- | --- | --- | --- | --- | --- | --- | --- | --- | --- | --- | --- | --- | --- |
| Module | Item | Factor 1 | | Factor 2 | |  | | Factor 1 | Factor 2 | Factor 3 | |  | | Factor 1 | Factor 2 | Factor 3 | Factor 4 | |  | | Factor 1 | Factor 2 | Factor 3 | Factor 4 | Factor 5 | |
| Language | H34 | -.019 | | **.260** | |  | | -.136 | .035 | **.422** | |  | | -.024 | .205 | -.167 | **.320** | |  | | -.154 | .287 | -.029 | .090 | **.349** | |
|  | H35 | **.480** | | .220 | |  |  | .306 | -.170 | **.785** | |  | | -.201 | .456 | .163 | **.554** | |  | | -.253 | **.581** | .090 | .412 | .245 | |
|  | H36 | **.359** | | .260 | |  |  | .234 | .119 | **.357** | |  | | .113 | -.163 | .411 | **.542** | |  | | .075 | -.133 | .099 | **.833** | .050 | |
|  | H37 | .179 | | **.361** | |  |  | .081 | .252 | **.267** | |  | | .198 | **.282** | -.037 | .172 | |  | | .217 | **.287** | -.154 | .191 | -.014 | |
|  | H38 | -.044 | | **.411** | |  |  | -.157 | .232 | **.358** | |  | | .228 | -.203 | .026 | **.513** | |  | | .080 | -.121 | .081 | .370 | **.418** | |
|  | H39 | **.463** | | -.040 | |  |  | **.431** | .056 | -.044 | |  | | .114 | -.126 | **.483** | .068 | |  | | .055 | -.151 | **.801** | -.032 | .405 | |
| Social Interaction | I40 | **.944** | | -.281 | |  | | **.902** | -.111 | -.091 | |  | | -.058 | -.027 | **.919** | .005 | |  | | .075 | .054 | **.575** | .313 | -.214 | |
|  | I41 | **.477** | | .278 | |  |  | .342 | .151 | **.358** | |  | | .021 | **.806** | -.078 | .088 | |  | | .021 | **.860** | -.025 | -.097 | .114 | |
|  | I42 | **1.004** | | -.385 | |  |  | **.982** | -.205 | -.120 | |  | | -.138 | .257 | **.733** | -.144 | |  | | .001 | .308 | **.543** | .038 | -.185 | |
|  | I43 | **.510** | | .139 | |  |  | **.422** | .118 | .168 | |  | | .017 | **.730** | .018 | -.093 | |  | | .101 | **.720** | -.033 | -.111 | -.115 | |
|  | I44 | **.490** | | .243 | |  |  | **.406** | .265 | .111 | |  | | .221 | **.414** | .161 | -.002 | |  | | .275 | **.428** | .097 | -.005 | -.011 | |
|  | I45 | -.120 | | **.402** | |  |  | -.173 | **.338** | .135 | |  | | **.316** | -.023 | -.124 | .158 | |  | | .215 | -.007 | .105 | -.081 | **.421** | |
| Flexibility | J46 | .360 | | **.410** | |  | | .269 | **.430** | .113 | |  | | .365 | **.493** | -.017 | -.039 | |  | | .374 | **.499** | .078 | -.185 | .143 | |
|  | J47 | .413 | | **.464** | |  |  | .331 | **.567** | .013 | |  | | **.554** | .104 | .260 | .068 | |  | | **.629** | .093 | .062 | .203 | -.075 | |
|  | J48 | -.298 | | **.798** | |  |  | -.393 | .**630** | .299 | |  | | **.548** | .062 | -.346 | .301 | |  | | **.512** | .033 | -.430 | .261 | .122 | |
|  | J49 | .286 | | **.550** | |  |  | .223 | **.771** | -.140 | |  | | **.746** | .119 | .116 | -.088 | |  | | **.778** | .086 | .107 | -.083 | .063 | |
|  | J50 | .236 | | **.555** | |  |  | .177 | **.774** | -.147 | |  | | **.773** | -.006 | .141 | -.044 | |  | | **.808** | -.039 | .074 | .011 | .028 | |

N = 295. All primary loadings are marked in bold.
