# Supplementary material for: The development of a brief screener for autism using item response theory
Source: BMC Psychiatry. 2019 Nov 4;19:337. doi: 10.1186/s12888-019-2333-y (PMC6829932; doi:10.1186/s12888-019-2333-y)
Supplement: Supplementary file 2 — Additional file 2: Table S2. Response frequencies. The table presents the response frequencies for each item in the developmental sample. [file 12888_2019_2333_MOESM2_ESM.docx]

**Response frequencies**

The following table includes the response frequencies for each item in the developmental sample.

**Table 1** Response frequencies.

| Item |  | Response categories | | |  | Missing |
| --- | --- | --- | --- | --- | --- | --- |
|  |  | No | Yes, to some extent | Yes |  |  |
| H34 |  | 91.2 | 3.4 | 5.2 |  | 0.2 |
| H35 |  | 96.7 | 2.2 | 0.8 |  | 0.3 |
| H36 |  | 94.7 | 3.1 | 1.9 |  | 0.3 |
| H37 |  | 95.9 | 2.3 | 1.4 |  | 0.3 |
| H38 |  | 87.9 | 7.4 | 2.4 |  | 2.3 |
| H39 |  | 87.9 | 7.2 | 2.7 |  | 2.2 |
| I40 |  | 97.8 | 1.4 | 0.6 |  | 0.2 |
| I41 |  | 94.0 | 4.1 | 1.7 |  | 0.2 |
| I42 |  | 97.5 | 1.7 | 0.5 |  | 0.2 |
| I43 |  | 93.0 | 5.0 | 1.8 |  | 0.2 |
| I44 |  | 91.4 | 5.3 | 1.5 |  | 1.7 |
| I45 |  | 75.2 | 18.6 | 6.0 |  | 0.2 |
| J46 |  | 89.1 | 7.5 | 3.2 |  | 0.2 |
| J47 |  | 96.0 | 2.8 | 1.0 |  | 0.2 |
| J48 |  | 96.1 | 1.8 | 2.0 |  | 0.2 |
| J49 |  | 88.9 | 8.1 | 2.8 |  | 0.2 |
| J50 |  | 86.6 | 9.5 | 3.7 |  | 0.2 |

N= 15,408
