# Supplementary material for: The development of a brief screener for autism using item response theory
Source: BMC Psychiatry. 2019 Nov 4;19:337. doi: 10.1186/s12888-019-2333-y (PMC6829932; doi:10.1186/s12888-019-2333-y)
Supplement: Supplementary file 3 — Additional file 3: Table S3. Cross tables, positive predictive value, negative predictive value and diagnostic odds ratio. Includes cross tables that present the numbers of true positive, false positive, true negative and false negative in the previous, predictive and total group for the ASD domain short form. The positive predictive value, negative predictive value and diagnostic odds ratio for the proposed cut-off values in the ASD domain short-form are also presented. [file 12888_2019_2333_MOESM3_ESM.docx]

**Cross tables**

The following tables present the numbers of true positive, false positive, true negative and false negative for ASD in the validation sample. The sample was divided into different groups based on age when the first ASD diagnosis was listed in the NPR: before (previous) or after (predictive) the A–TAC interview. The analyses were also conducted in a collapsed fashion, including the total sample.

The rows in the tables present the number of screen-positive or screen-negative subjects in the ASD domain for each proposed cut-off value. The columns present the number of subjects with or without a registered disorder in NPR.

**Table 1** Previous.

| **Cut-off: 0.5** | ASD | No ASD | **Cut-off: 1.5** | ASD | No ASD |
| --- | --- | --- | --- | --- | --- |
| Screen-positive | 74 | 1534 |  | 51 | 236 |
| Screen-negative | 4 | 13641 |  | 27 | 14939 |

N = 15,253 (missing = 237)

**Table 2** Predictive.

| **Cut-off: 0.5** | ASD | No ASD | **Cut-off: 1.5** | ASD | No ASD |
| --- | --- | --- | --- | --- | --- |
| Screen-positive | 67 | 1541 |  | 32 | 255 |
| Screen-negative | 62 | 13583 |  | 97 | 14869 |

N = 15,253 (missing = 237)

**Table 3** Total.

| **Cut-off: 0.5** | ASD | No ASD | **Cut-off: 1.5** | ASD | No ASD |
| --- | --- | --- | --- | --- | --- |
| Screen-positive | 141 | 1467 |  | 83 | 204 |
| Screen-negative | 66 | 13579 |  | 124 | 14842 |

N = 15,253 (missing = 237)

**Positive predictive value, negative predictive value and diagnostic odds ratio**

Table 4 includes the positive predictive value, negative predictive value and diagnostic odds ratio for the proposed cut-off values in the ASD domain short-form. The calculations were conducted in the validation sample.

**Table 4** Positive predictive value, negative predictive value and diagnostic odds ratio.

|  |  | PPV | NPV | DOR |
| --- | --- | --- | --- | --- |
| Previous | low | .05 | .999 | 165 |
|  | high | .18 | .998 | 120 |
| Predictive | low | .04 | .995 | 10 |
|  | high | .11 | .994 | 19 |
| Total | low | .09 | .995 | 20 |
|  | high | .29 | .992 | 49 |

N = 15,253

For clarity reasons, NPV is reported with three decimals.
